# Malignant Mesothelioma and Delivery of Polyphenols

**DOI:** 10.3390/nu8060335

**Published:** 2016-06-02

**Authors:** Karen S. Bishop, Andrea J. Braakhuis, Lynnette R. Ferguson

**Affiliations:** 1 Auckland Cancer Society Research Center, FM & HS, University of Auckland, Private Bag 92019, Auckland 1142, New Zealand; 2 Discipline of Nutrition and Dietetics, FM & HS, University of Auckland, Private Bag 92019, Auckland 1142, New Zealand; a.braakhuis@auckland.ac.nz (A.J.B.); l.ferguson@auckland.ac.nz (L.R.F.)

Malignant Mesothelioma (MM) is a rare form of cancer that affects the thin cell wall lining of the body’s internal organs and structures. MM has a particularly poor outcome following standard treatment options. MM is found in the pleura, the peritoneum, and more rarely in the heart. MM is known only to be caused by exposure to asbestos fibres either directly, or through someone who was exposed, and this risk may be modified by genotype [1]. The disease develops through a multistep process resulting from chronic inflammation, DNA damage and dysregulation of the immune system. Benvenuto and colleagues from the University of Rome recently published an article of great public interest in the *Nutrients* Special Issue: Polyphenols for Cancer Treatment or Prevention, wherein they present current knowledge on the properties of polyphenols and protective effects against asbestos-mediated MM [2].

Polyphenols are commonly found in edible plants, are known to improve the immune function, reduce chronic inflammation [2], modify aberrant intraperitoneal cytokine levels [3] and reduce growth of cancer cells [2]. Benvenuto *et al.* list the various subclasses of polyphenols and their sources, and in addition, highlight the fact that a reduction in chronic inflammation may be the key to the prevention and/or stunted progression of MM [2].

Interestingly, Benvenuto *et al.* [2] provide a “profile” of up and down regulated cytokines for healthy subjects *vs*. asbestos exposed *vs*. MM patients. Upon further refinement, such profiles may be useful as a biomarker for MM risk and progression.

Despite the health benefits associated with polyphenols, the bioavailability of many polyphenol bioactives limits their effect. Problems with poor absorption, fast-metabolism and food preparation techniques, amongst others, remain to be solved. Importantly, Benvenuto *et al.* [2] have summarised the relevant published literature and suggest administering polyphenols to the serous cavity, so as to avoid problems associated with bioavailability and to deliver a clinically relevant dose to the tumour site. The location of MM tumours makes intratumoral administration feasible.

Bioavailability is a common problem whether we are considering the action of drugs or the clinical application of bioactives in foods. Granja and colleagues, from the University of Porto, Portugal, in a recent publication in the same *Nutrients* Special Issue, also recognise the need to improve stability and bioavailability of food bioactives that have shown promising anti-cancer activity *in vitro*. They reviewed published data and summarised various delivery systems for the polyphenol (−)-Epigallocatechin-3-gallate (EGCG). EGCG is most commonly associated with green tea, and is believed to work in synergy with various anti-cancer drugs [4]. Granja *et al.* [4] provide an overview of the nanotechnologies employed to overcome the poor pharmacokinetic and pharmacodynamics of this promising anti-cancer agent. In particular, gold nanoparticles; biodegradable, polymeric nanoparticles, functionalised with cell surface specific antibodies; as well as nanoliposomes [4], hold much promise for the delivery or co-delivery (with FDA approved anti-cancer drugs) of polyphenols as anti-cancer agents. Further research is required, but such an approach is likely to solve many of the challenges surrounding absorption and bioavailability of polyphenols to aid in the prevention and treatment of numerous cancers, as well as their treatment related side-effects. 

## References

[1] Tunesi S., Ferrante D., Mirabelli D., Andorno S., Betti M., Fiorito G., Guarrera S., Casalone E., Neri M., Ugolini D. (2015). Gene–asbestos interaction in malignant pleural mesothelioma susceptibility. Carcinogenesis.

[2] Benvenuto M., Mattera R., Taffera G., Giganti M.G., Lido P., Masuelli L., Modesti A., Bei R. (2016). The Potential Protective Effects of Polyphenols in Asbestos-Mediated Inflammation and Carcinogenesis of Mesothelium. Nutrients.

[3] Miller J.M., Thompson J.K., MacPherson M.B., Beuschel S.L., Westbom C.M., Sayan M., Shukla A. (2014). Curcumin: A Double Hit on Malignant Mesothelioma. Cancer Prev. Res. (Phila Pa).

[4] Granja A., Pinheiro M., Reis S. (2016). Epigallocatechin Gallate Nanodelivery Systems for Cancer Therapy. Nutrients.

